# Focal ^18^F‐FDG uptake predicts progression of pre‐invasive squamous bronchial lesions to invasive cancers

**DOI:** 10.1111/1759-7714.14815

**Published:** 2023-02-18

**Authors:** Illaa Smesseim, Robert A. van Boerdonk, Chris Dickhoff, David J. Heineman, Max R. Dahele, Teodora Radonic, Idris Bahce, Simone P. Rauh, Emile F. I. Comans, Hans (J. M. A.) Daniels

**Affiliations:** ^1^ Department of Pulmonary Diseases Amsterdam University Medical Center, Location Free University Medical Center Amsterdam The Netherlands; ^2^ Department of Pathology Amsterdam University Medical Center Amsterdam The Netherlands; ^3^ Department of Cardiothoracic Surgery Amsterdam University Medical Center Amsterdam The Netherlands; ^4^ Department of Radiotherapy Amsterdam University Medical Center Amsterdam The Netherlands; ^5^ Department of Epidemiology and Biostatistics Amsterdam University Medical Center Amsterdam The Netherlands; ^6^ Department of Radiology and Nuclear Medicine Amsterdam University Medical Center Amsterdam The Netherlands

**Keywords:** endobronchial, lung cancer, lung carcinoma, PET‐scan, pre‐invasive lesions

## Abstract

**Introduction:**

Pre‐invasive squamous lesions of the central airways can progress into invasive lung cancers. Identifying these high‐risk patients could enable detection of invasive lung cancers at an early stage. In this study, we investigated the value of ^18^F‐fluorodeoxyglucose (^18^F‐FDG) positron emission tomography (PET) scans in predicting progression in patients with pre‐invasive squamous endobronchial lesions.

**Methods:**

In this retrospective study, patients with pre‐invasive endobronchial lesions, who underwent an ^18^F‐FDG PET scan at the VU University Medical Center Amsterdam, between January 2000 and December 2016, were included. Autofluorescence bronchoscopy (AFB) was used for tissue sampling and was repeated every 3 months. The minimum and median follow‐up was 3 and 46.5 months. Study endpoints were the occurrence of biopsy proven invasive carcinoma, time‐to‐progression and overall survival (OS).

**Results:**

A total number of 40 of 225 patients met the inclusion criteria of which 17 (42.5%) patients had a positive baseline ^18^F‐FDG PET scan. A total of 13 of 17 (76.5%) developed invasive lung carcinoma during follow‐up, with a median time to progression of 5.0 months (range, 3.0–25.0). In 23 (57.5%) patients with a negative ^18^F‐FDG PET scan at baseline, 6 (26%) developed lung cancer, with a median time to progression of 34.0 months (range, 14.0–42.0 months, *p* < 0.002). With a median OS of 56.0 months (range, 9.0–60.0 months) versus 49.0 months (range, 6.0–60.0 months) (*p* = 0.876) for the ^18^F‐FDG PET positive and negative groups, respectively.

**Conclusions:**

Patients with pre‐invasive endobronchial squamous lesions and a positive baseline ^18^F‐FDG PET scan were at high‐risk for developing lung carcinoma, highlighting that this patient group requires early radical treatment.

## Graphical abstract

Patients with pre‐invasive endobronchial squamous lesions and a positive baseline ^18^F‐fluorodeoxyglucose positron emission tomography scan were at high‐risk for developing lung carcinoma, highlighting that this patient group requires early radical treatment.

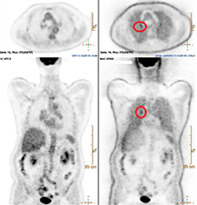

## INTRODUCTION

Lung cancer is the leading cause of cancer‐related death worldwide among both men and women.^1^ Approximately 85% of lung cancers have non–small cell histology (non–small cell lung cancers [NSCLC]), with adenocarcinoma being the predominant subtype. These tumors are typically located in the lung parenchyma. In contrast, squamous cell carcinoma (SCC) accounts for 20% to 30% of NSCLCs, and frequently originates from the central airway.^1^ Although in recent surgical series, a shift towards more peripherally located SCC has been observed.^2^, ^3^ Nevertheless, a conservative estimate is that ~15%–20% of all lung cancers are central type squamous cancers, which therefore, constitutes a major worldwide cancer burden.^4^, ^5^ SCC originates from pre‐invasive lesions of the airway epithelium.^6^ When such pre‐invasive lesions are identified, they need intermittent surveillance using autofluorescence bronchoscopy (AFB), which exploits the spectral differences in fluorescence and absorption properties of normal and dysplastic bronchial epithelium, resulting in improved ability of early detection of centrally located pre‐invasive endobronchial squamous lesions.^7^, ^8^, ^9^, ^10^, ^11^ Although the diagnostic role of AFB has decreased in recent years, the technique has proved its usefulness in detecting paraneoplastic lesions in prior studies.^11^, ^12^

In our institute, AFB is routinely used to localize and map the mucosal extent of pre‐invasive lesions within the bronchial tree of patients perceived to be at high‐risk (based on symptoms, smoking status, cancer history, and chronic obstructive pulmonary disease [COPD] status) of (second) primary lung cancer or cancer recurrence.^5^ However, when analyzing this cohort of patients, it appears that only a minority of these pre‐invasive endobronchial lesions progress to invasive lung cancer.^5^ A strategy of performing bronchoscopic surveillance on all individuals with pre‐invasive lesions is, therefore, inefficient and undesirable. Instead, (predictive) biomarkers are required to select patients at high‐risk of progression. We and others have shown that molecular techniques can be used to identify high‐risk pre‐invasive lesions more precisely.^13^, ^14^, ^15^ However, these techniques require tissue, can be time consuming, are costly, and carry the risk of sampling error.

Imaging techniques such as ^18^F‐Fluorodeoxyglucose (^18^F‐FDG) positron emission tomography (PET) might be a less invasive alternative for predicting progression to cancer.^16^ Two small studies report ^18^F‐FDG PET to be able to discriminate invasive from pre‐invasive, radiographically occult lesions^17^ and that focal ^18^F‐FDG uptake can identify carcinoma in situ (CIS) lesions at high risk of progression to cancer.^16^

In the present study, we report the results and predictive value of ^18^F‐FDG PET scans in patients with pre‐invasive endobronchial lesions who were followed in a surveillance program. The aim was to gain more insight into the role of ^18^F‐FDG PET scans in the evaluation of pre‐invasive endobronchial lesions and with particular focus on its potential role as an imaging biomarker for predicting progression to invasive carcinomas.

## MATERIALS AND METHODS

### Study population

We performed a single‐institution retrospective study, including all subsequent patients with pre‐invasive endobronchial lesions that were referred to our center for diagnosis and staging. Between January 2000 and December 2016, a total of 225 patients were referred and underwent AFB. We selected patients who met the following inclusion criteria: (1) presence of one or more pre‐invasive endobronchial lesions at initial AFB examination, including hyperplasia, squamous metaplasia, mild, moderate, and severe dysplasia and carcinoma in situ as confirmed by histology, and (2) had undergone a ^18^F‐FDG PET scan before the first AFB. We excluded patients with a (1) follow up of less than 3 months after AFB and (2) invasive endobronchial carcinoma confirmed by histology. The following data were extracted: age, sex, history of lung cancer, smoking history, COPD status, and endobronchial histology at baseline. This study was reviewed and approved by the ethics committee of the Amsterdam University Medical Centers, location Vrije Universiteit Medisch Centrum (VUmc). The participants provided their written informed consent to participate in this study.

### Study endpoints

The primary endpoint was the occurrence of biopsy proven invasive carcinoma. The following types of lung cancer were defined: (1) site‐specific progression, defined as lung cancer originating from a pre‐invasive endobronchial lesion, and (2) interval cancer, defined as lung cancer at a previously non‐suspicious endobronchial site. Other endpoints included time‐to‐progression of a pre‐invasive lesion to invasive cancer and overall survival (OS), defined as the time from inclusion until death from any cause. Routine histological assessment of hematoxylin and eosin (H&E)‐stained formalin‐fixed and paraffin‐embedded (FFPE) tissue sections was performed in accordance to the World Health Organization/International Association for the Study of Lung Cancer (WHO/IASLC) criteria for histological classification of lung and pleural tumors.^18^, ^19^ The patients were grouped into a low‐grade disease group (LGD) (including hyperplasia, squamous metaplasia, and mild and moderate dysplasia) and high‐grade disease group (HGD) (including severe dysplasia and carcinoma in situ).

One experienced nuclear medicine physician reviewed all ^18^F‐FDG PET images that were made at baseline and dichotomously scored the presence or absence of focal FDG uptake, defined as an intensity greater than the physiological uptake in the surrounding tissue.^20^ The standardized uptake value (SUV) was measured for lesions in patients scanned using a hybrid ^18^F‐FDG PET‐CT scanner, where both computed tomography (CT)‐based attenuation‐corrected (CTAC) and non‐attenuation‐corrected PET (NAC‐PET) images were available. The SUV could not be measured in patients that were included before 2007 because these patients only had a PET‐scan without a CT scan. In these patients, focal uptake was defined as well‐circumscribed areas of increased tracer uptake relative to the surrounding structures, defined by an experienced nuclear medicine physician.^20^ Patients that were included before 2007 only had NAC‐PET images.

#### Autofluorescence bronchoscopy

Experience with detecting abnormal tissue by using AFB is very important. AFB was performed by expert bronchoscopists (J.M.A.D. and Tom G. Sutedja) under local anesthesia. To increase the reliability of the biopsies, multiple biopsies were taken in a standardized fashion from suspicious hypofluorescent areas. We did not use bite‐on‐bite or stacked biopsy specimens because we have the experience that with sufficient pressure on the tissue, adequate samples can be acquired. To that end we opened the biopsy forceps, pulled it back against the distal tip of the bronchoscope, and pushed the open forceps in the tissue by pushing the bronchoscope down. To prevent cross‐contamination separate forceps were used for each biopsy site. Before histological examination the biopsies were FFPE.

### Statistical analysis

SPSS Statistics (version 20.0) was used to perform statistical analyses. Two‐sided *p*‐values <0.05 were considered statistically significant in all our tests. Differences between groups were estimated using Fisher's exact and the Mann–Whitney *U* test. The Kaplan–Meier method was used for survival analysis. Multivariate Cox regression analysis was used to detect potential risk determinants.

## RESULTS

### Patient characteristics

**FIGURE 1:**
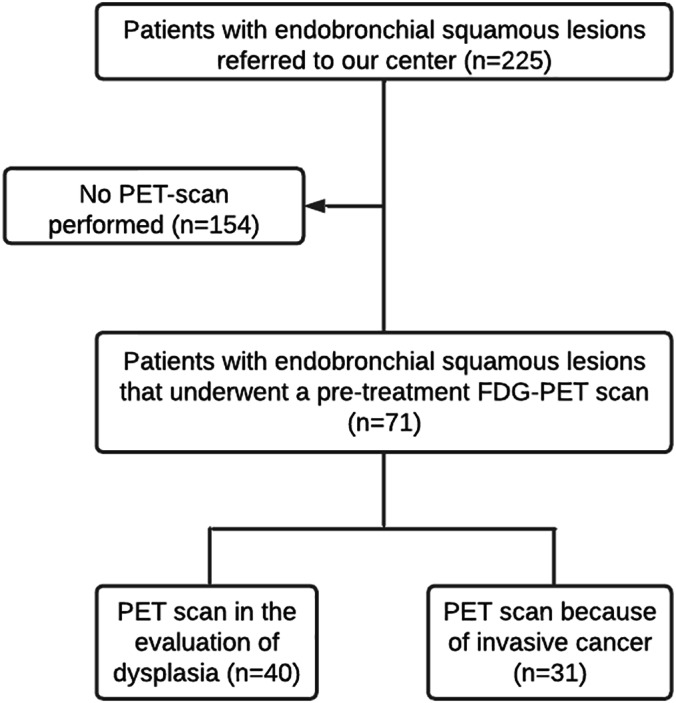
Patient selection.

Of 225 patients, 71 had an ^18^F‐FDG PET scan at baseline and a follow up period of at least 3 months. Thirty‐one of these 71 patients were excluded because of histologically confirmed invasive carcinoma at baseline, resulting in 40 patients eligible for analysis (Figure 1). Table 1 summarizes the baseline characteristics: 82.5% of patients were male and the median age was 68.0 (52.0–82.0) years, 65% had a history of lung cancer. Baseline characteristics were comparable for patients in the ^18^F‐FDG PET‐positive and the ^18^F‐FDG PET‐negative group. All patients underwent surveillance with repeated AFB examinations, biopsies, and CT scans every 3 months. The median follow up was 46.5 (IQR, 25.3–80.0) months.

**TABLE 1:** Baseline characteristics of the patient population.

	PET‐positive group (*n* = 17)	PET‐negative group (*n* = 23)	*p*‐value
Age (median in years, range)	69.0 (52.0–82.0)	67.0 (53.0–76.0)	0.338
Sex (*n*, %)			0.101
Male	16 (94)	17 (74)	
Female	1 (6)	6 (26)	
Smoking status (*n*, %)			0.653
Never	0	1 (4)	
Former	9 (53)	10 (43)	
Current	8 (47)	12 (53)	
Histology pre‐invasive lesion at baseline (*n*, %)			0.228
LGD	7 (41)	14 (61)	
HGD	10 (59)	9 (39)	
COPD status (*n*, %)			0.295
COPD	9 (53)	16 (70)	
No COPD	8 (47)	7 (30)	
History of lung cancer (*n*, %)			0.175
Yes	9 (53)	17 (74)	
No	8 (47)	6 (26)	
Pack years (median in years, range)	45.0 (20.0–60.0)	41.0 (0.0–120.0)	0.977

Abbreviations: PET, positron emission tomography; COPD, chronic obstructive pulmonary disease; HGD, high grade dysplasia, severe dysplasia and carcinoma in situ; LGD, low grade dysplasia, including hyperplasia, squamous metaplasia, mild and moderate dysplasia.

### Lesional FDG uptake

Seventeen of 40 patients with a pre‐invasive lesion demonstrated increased FDG uptake. As ^18^F‐FDG PET‐scans before 2007 were not combined with a low dose CT scans, the SUV‐value could only be measured for 3 of 17 patients with a positive ^18^F‐FDG PET‐CT scan. The SUV values were SUV_mean_ lesion 5.3, SUV_mean_ liver 2.0 (SUV_max_ lesion 6.4), SUV_mean_ lesion, 2.9, SUVmean liver 2.5 (SUV_max_ lesion 3.3) and SUV_mean_ lesion 4.9, SUV_mean_ liver 2.5 (SUV_max_ lesion 6.2).

### Progression to invasive lung cancer

**FIGURE 2:**
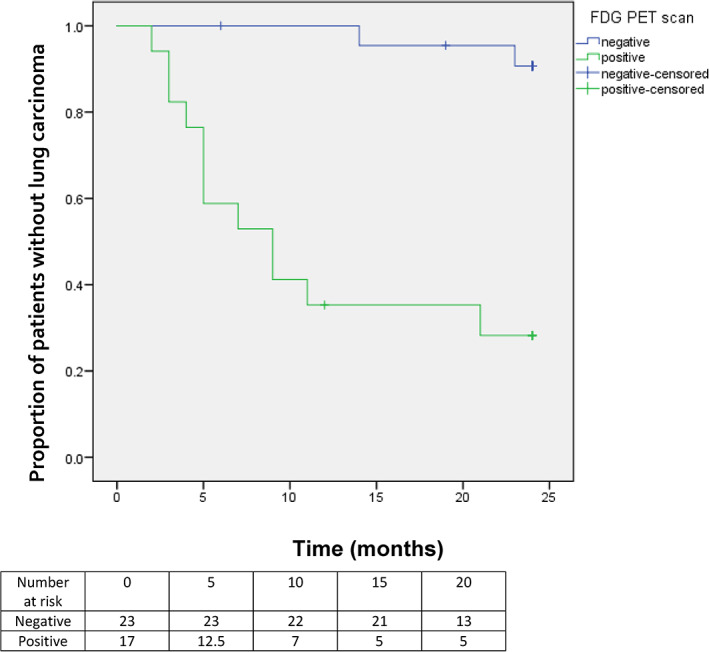
Progression free survival of patients with pre‐invasive lesions with a positive and negative FDG‐PET scan (*n* = 40; HR, 14.72; *p* = 0.002). FDG‐PET, fluorodeoxyglucose positron emission tomography; HR, hazard ratio.

Invasive lung cancer was detected during follow up with AFB in 13 of 17 (76.5%) patients in the ^18^F‐FDG PET‐positive group and in 6 of 23 (26.1%) patients in the ^18^F‐FDG PET‐negative group (odds ratio [OR], 9.2; 95% confidence interval [95% CI], 2.1–39.5, *p* = 0.002) (Table 2). Median time to progression to invasive lung cancer was 5.0 months (range, 3.0–25.0) in the ^18^F‐FDG PET‐positive group and 34.0 months (14.0–42.0 months) in the ^18^F‐FDG PET‐negative group (*p* = 0.002; hazard ratio [HR], 14.72) (Figure 2). Site‐specific progression occurred in eight of 18 patients from the ^18^F‐FDG PET‐positive group (47%) and two of 23 patients in the ^18^F‐FDG PET‐negative group (8.7%) (OR, 9.3; 95% CI, 1.6–52.9). Interval cancer occurred in 5 patients of the ^18^F‐FDG PET‐positive group (29%) and four patients of the ^18^F‐FDG PET‐negative group (17%) (OR, 2.0; 95% CI, 0.4–8.9). Seven of 17 patients (41%) in the PET‐positive group had low‐grade pre‐invasive lesions at baseline.

**TABLE 2:** Outcomes of progression of pre‐invasive squamous bronchial lesions to invasive cancers.

	PET‐positive group (*n* = 17)	PET‐negative group (*n* = 23)	OR [95% CI]	*p* value
Progression overall	13 (76.5%)	6 (26.1%)	9.2 [2.1–39.5]	0.002
Site specific progression	8 (47.1%)	2 (8.7%)	9.3 [1.6–52.9]	0.009
Interval cancer	5 (29.4%)	4 (17.4%)	2.0 [0.4–8.9]	0.456

Abbreviations: PET, positron emission tomography; OR, odds ratio; CI, confidence interval.

### Survival outcomes

**FIGURE 3:**
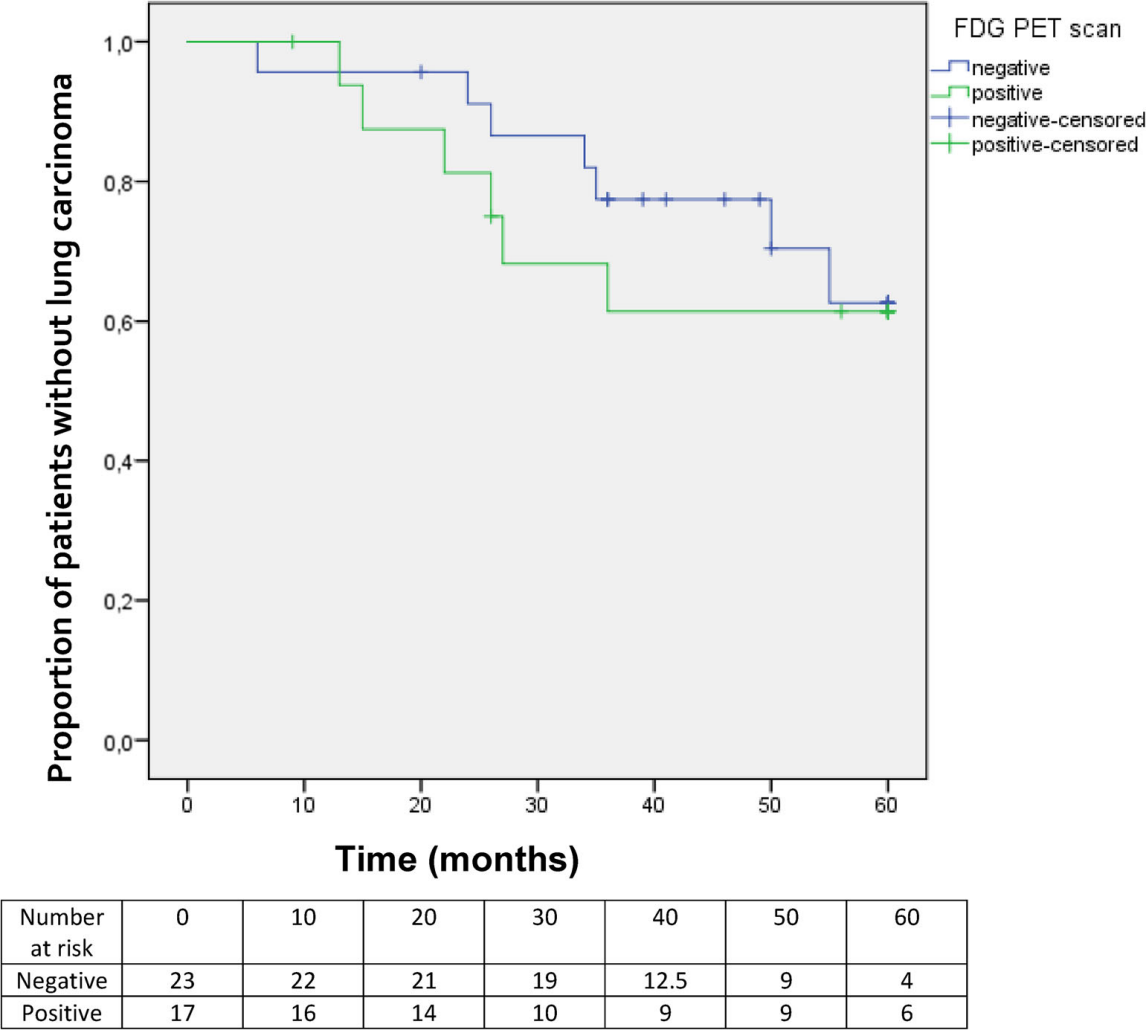
Overall survival of patients with pre‐invasive lesions with a positive and negative ^18^F‐FDG‐PET with no lung carcinoma at baseline (*n* = 40; HR, 1.31; *p* = 0.876). ^18^F‐FDG‐PET ^18^F‐fluorodeoxyglucose positron emission tomography; HR, hazard ratio.

A total of 14 of 40 patients (35%) died with a median follow up of 25.5 (interquartile range [IQR], 20.3–39.5) months, of which 12 were lung cancer related. The median overall survival was 56.0 months (range, 9.0–60.0 months) in the ^18^F‐FDG PET‐positive group and 49.0 months (range, 6.0–60.0 months) in the ^18^F‐FDG PET‐negative group (*p* = 0.876; HR, 1.31) (Figure 3). One patient in the ^18^F‐FDG PET‐negative group died 6 months after inclusion (unrelated to lung cancer). Multivariate Cox regression analysis that controlled for additional risk factors revealed no independent risk determinants for overall survival. There was also no significant difference observed in OS before and after crossing of the OS curves at 13.0 months, the assumption of proportionality was not violated.

## DISCUSSION

In this study, we showed that patients with pre‐invasive endobronchial lesions and a positive ^18^F‐FDG PET scan had a nearly 3‐fold higher risk for developing lung cancer and had a significantly shorter time to progression than those with a negative ^18^F‐FDG PET scan (HR, 14.7). Our results suggest that a positive baseline ^18^F‐FDG PET scan in these patients is a predictor of progression to invasive cancer and that close monitoring or initiation of radical cancer treatment is required in this group of patients.

The cohort of patients with pre‐invasive central SCC lesions had a remarkably long follow up period (median, 46.5 months). These findings are highly relevant for the routine clinical practice as the optimal management of these pre‐invasive lesions is still unclear. Therefore, qualified biomarkers that predict progression to invasive cancers are urgently needed. Our findings add to the clinical biomarker validation effort of ^18^F‐FDG PET for predicting progression to invasive cancers. In the ^18^F‐FDG PET‐negative group, 6 of 23 (26.1%) patients developed invasive cancer, so we should also state that a single baseline ^18^F‐FDG PET scan is inadequate for identifying patients who will not develop cancer. Whether repeat ^18^F‐FDG PET scanning would improve accuracy, has not been tested.

Our findings are consistent with previously reported data. To our knowledge, only two studies investigated the role of ^18^F‐FDG PET scans in assessing the risk of developing lung cancer in patients with pre‐invasive endobronchial lesions. Pasic et al.^17^ reported on 24 patients with pre‐invasive lesions (*n* = 13) and early SCC (*n* = 11). In this study, the ^18^F‐FDG PET scan findings were correlated with histology taken at AFB. ^18^F‐FDG PET was true positive for invasive cancer in 8 of 11 cases and true negative (severe dysplasia or less on biopsy) in 11 of 13 cases corresponding to a sensitivity/specificity of 73/85% and a positive/negative predictive value of 80/79%. Although the study by Pasic et al.^17^ was from our institution, only a limited number of patients were included and analyzed in both studies, because patients with early squamous cancer were excluded from the current analysis, and there were only 12 (12/40 = 30%) patients from the current study with pre‐invasive lesions in the period 2000 to 2003 (the period of the Pasic study). In addition, Pasic et al.^17^ investigated the correlation between FDG uptake and histology at the same point in time, whereas the current analysis investigated FDG uptake as a predictor for progression to cancer. Fraioli et al.^16^ investigated a group of 44 patients with pre‐invasive endobronchial lesions that underwent ^18^F‐FDG PET scans within 6 weeks of the AFB examination. They observed no focal FDG uptake in patients with any grade of dysplasia. In patients with CIS, 88% (7/8) of patients with focal FDG uptake developed invasive cancer versus 29% (6/21) of patients with no FDG uptake. This exclusivity of focal FDG uptake for CIS lesions differs from our results. One explanation for this finding could be interobserver variability in the grading of these lesions by the pathologist. However, interobserver variability of the WHO/IASLC classification for pre‐invasive bronchial lesions ranges from moderate to good (mean weighted κ statistics of 0.51–0.66), which makes it very unlikely that all low‐grade lesions with focal ^18^F‐FDG uptake were graded incorrectly.^21^ Sampling error could be another explanation for focal FDG uptake. Although biopsies of the bronchial mucosa show premalignant disease, focal FDG uptake might be explained by a small volume of extraluminal, invasive tissue. Radial Endobronchial Ultrasound Bronchoscopy (EBUS) can be used to assess the depth of invasion in or beyond the bronchial wall, but this technique was not used in our cohort.^22^, ^23^ Nonetheless, independent of the discussion about the exclusivity of ^18^F‐FDG PET, the observed focal FDG uptake and subsequent progression to cancer in patients with low grade lesions is in line with earlier observations in our cohort that patients with low grade lesions can harbor multiple genetic aberrations and can progress to lung cancer in a relatively short time span.^5^, ^14^, ^15^ A prospective study correlating FDG uptake with genetic aberrations in premalignant lesions could shed more light on this issue.

FDG‐uptake in tumor cells is variable and influenced by factors such as hypoxia, growth rate, histology and Glut‐1 expression (a cell membrane glucose transporter).^24^ Brown et al.^24^ found that Glut‐1 concentration is upregulated and FDG uptake is very high in SCC compared with the majority of adenocarcinomas.^25^ SCC is traditionally known to arise centrally (66%–90%) and is therefore, more likely to ultimately develop significant airway obstruction, leading to atelectasis and pneumonia distal to a bronchial obstruction. This may result in false–positive focal FDG uptake both in the lung parenchyma and in hilar and/or mediastinal lymph nodes. Our advice would, therefore, be to exclude lymph node metastases with EBUS/EUS in these patients. In our study, the small lesions did not cause total obstruction of the central airways yet. Therefore, in this clinical context, focal uptake in the central airways should be considered suspicious for invasive SCC.

**FIGURE 4:**
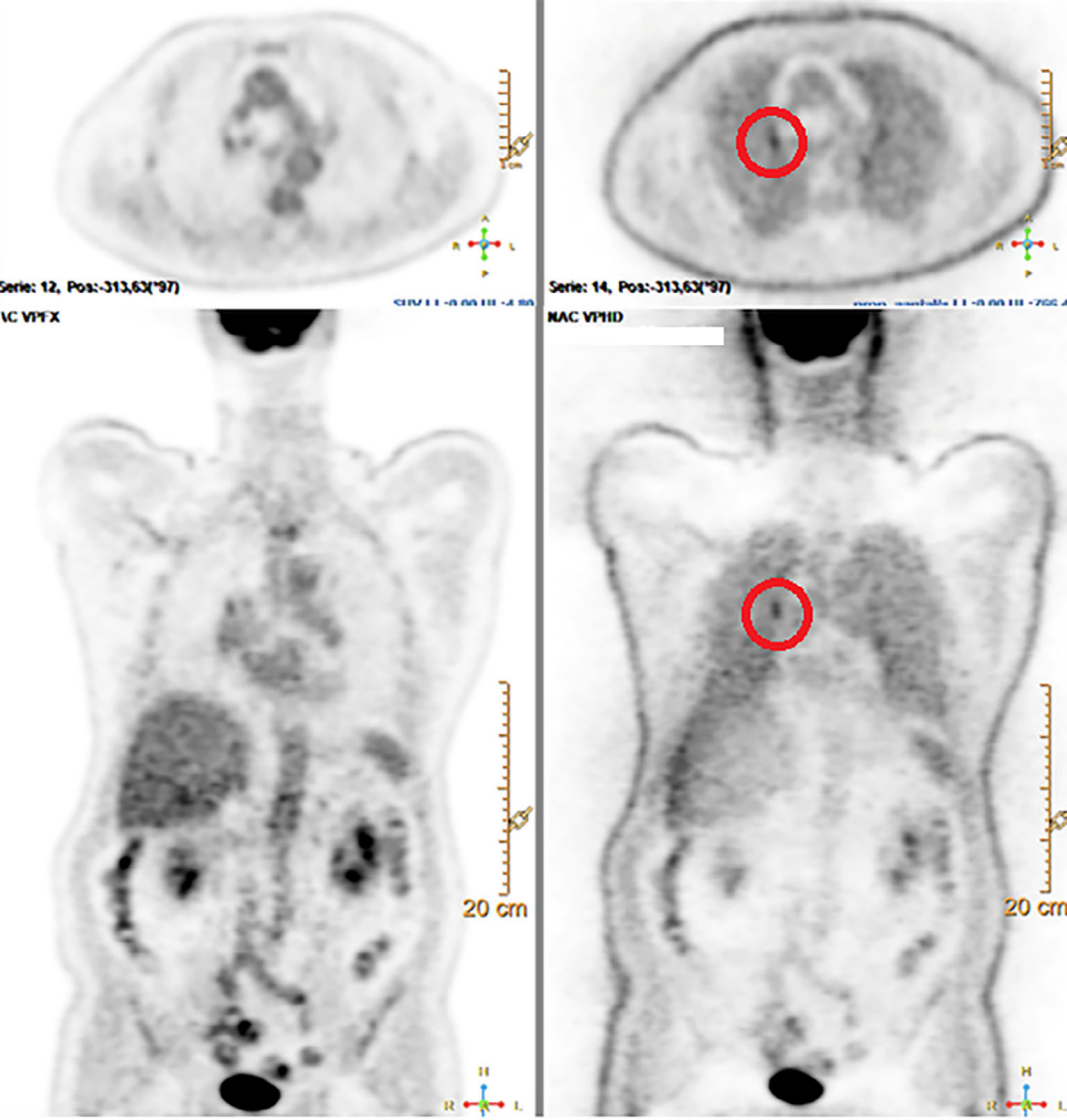
Example of axial and coronal view of CTAC PET on the left and NAC PET on the right of a patient with a pre‐invasive lesion in the right upper lobe that was initially missed on the CTAC images. CTAC, computed tomography‐based attenuation‐corrected; PET, positron emission tomography; NAC, non‐attenuation‐corrected.

Maintas et al.^26^ showed that peripheral malignant lesions were better visualized on the NAC images in comparison to the CTAC images, especially in the basal lung fields. Mismatch in the co‐registration between the CT‐based location of a small lung nodule and the ^18^F‐FDG PET‐based location of the focally increased FDG uptake causes underestimation of the measured local CT‐attenuation because of the calculation of the SUV by incorrect Hounsfield Unit values. NAC images are free of these potential artifacts. It is, therefore, essential for the accurate detection of small lesions in the central airways to evaluate both NAC and CTAC images, which is illustrated in Figure 4. This figure show a pre‐invasive lesion located centrally in the right upper lobe on the NAC image that was initially missed on the CTAC images, but detected on NAC images from a follow up ^18^F‐FDG PET‐scan 5 months later. In addition, caution is advocated regarding the use of SUV measurements, as partial volume effects (defined as the loss of activity in small lesions because of limited resolution of the imaging system) may lead to underestimation of the SUV value.^27^

In this respect, it is important to emphasize that the visual evaluation of ^18^F‐FDG PET images remains the standard in the absence of diagnostic criteria that are generally accepted worldwide. As an alternative in some studies the FDG uptake on CTAC images is visually compared to liver uptake to discriminate between a ^18^F‐FDG PET‐positive and ^18^F‐FDG PET‐negative lesion, as is now the accepted standard in the evaluation of metabolic response in aggressive lymphoma patients (the so called Deauville score).^28^ As illustrated in Figure 4, the focal uptake in the central pulmonary lesion does not exceed liver activity on visual analysis.

There are several limitations that deserve to be mentioned. This was a retrospective study that included patients with pre‐invasive lesions referred to a tertiary center for analysis and surveillance. AFB relies on differences in the wavelength of emitted visible light between normal and dysplastic bronchial epithelium, which has markedly improved earlier detection of centrally located pre‐invasive endobronchial lesions. Although this technique is more sensitive than white light flexible bronchoscopy, it is still prone to sampling error. The decision to acquire an ^18^F‐FDG PET‐scan was made by the treating pulmonologist and was based on a high estimated risk for developing lung cancer, taking into account smoking status, cancer history, and COPD status. This introduces referral and selection bias. Because of the retrospective design of our study, we could only select a small selection of all patients who were referred to our institution with pre‐invasive lesions and had a pre‐AFB PET‐scan. Further prospective research is needed in which PET‐scans will be performed for all patients referred to our institution for pre‐invasive lesions, this will help with selecting a larger group of eligible patients. Another limitation of our study is the differences in PET‐protocols in the time span of the study. For example, the SUV‐values could only be measured for PET‐scans after 2007 because PET‐scans could not be combined with a CT scan before 2007.

## CONCLUSION

Patients with pre‐invasive endobronchial squamous lesions and focal FDG uptake on ^18^F‐FDG PET (CT) are at high‐risk of progression to invasive lung cancers. The time‐to‐progression in the ^18^F‐FDG PET‐positive group is significantly shorter than the ^18^F‐FDG–negative group. This suggests that patients with a positive scan merit a more aggressive approach with either close surveillance or early radical treatment (e.g., surgery, radiotherapy). Further research is needed to investigate whether early treatment of patients with pre‐invasive lesions and focal FDG uptake improves outcome.

## CONFLICT OF INTEREST STATEMENT

The authors declare no conflicts of interest.
